# Supplementary material for: In silico design and immunoinformatics analysis of a universal multi-epitope vaccine against monkeypox virus
Source: PLoS One. 2023 May 23;18(5):e0286224. doi: 10.1371/journal.pone.0286224 (PMC10205007; doi:10.1371/journal.pone.0286224)
Supplement: S1 Data — (PDF) [file pone.0286224.s001.pdf]

Multiple sequence alignment of cell surface-binding protein.

```
CAA66449.1          MPQQLSPINIETKKAISDARLKTLDIHYNESKPTTIQNTGKLVRINFKGGYISGGFLPNE   60
WDQ84781.1          MPQQLSPINIETKKAISDARLKTLDIHYNESKPTTIQNTGKLVRINFKGGYISGGFLPNE   60
WBT04084.1          MPQQLSPINIETKKAISDARLKTLDIHYNESKPTTIQNTGKLVRINFKGGYISGGFLPNE   60
UYW97776.1          MPQQLSPINIETKKAISDARLKTLDIHYNESKPTTIQNTGKLVRINFKGGYISGGFLPNE   60
WFD73904.1          MPQQLSPINIETKKAISDARLKTLDIHYNESKPTTIQNTGKLVRINFKGGYISGGFLPNE   60
WDO63050.1          MPQQLSPINIETKKAISDARLKTLDIHYNESKPTTIQNTGKLVRINFKGGYISGGFLPNE   60
UZT62701.1          MPQQLSPINIETKKAISDARLKTLDIHYNESKPTTIQNTGKLVRINFKGGYISGGFLPNE   60
UZV08183.1          MPQQLSPINIETKKAISDARLKTLDIHYNESKPTTIQNTGKLVRINFKGGYISGGFLPNE   60
AAU01309.1          MPQQLSPINIETKKAISDARLKTLDIHYNESKPTTIQNTGKLVRINFKGGYISGGFLPNE   60
AUW64186.1          MPQQLSPINIETKKAISDARLKTLDIHYNESKPTTIQNTGKLVRINFKGGYISGGFLPNE   60
WFJ79428.1          MPQQLSPINIETKKAISDARLKTLDIHYNESKPTTIQNTGKLVRINFKGGYISGGFLPNE   60
WCC70104.1          MPQQLSPINIETKKAISDARLKTLDIHYNESKPTTIQNTGKLVRINFKGGYISGGFLPNE   60
UYD49617.1          MPQQLSPINIETKKAISDARLKTLDIHYNESKPTTIQNTGKLVRINFKGGYISGGFLPNE   60
AAY97104.1          MPQQLSPINIETKKAISDTRLKTLDIHYNESKPTTIQNTGKLVRINFKGGYISGGFLPNE   60
YP_010377102.1      MPQQLSPINIETKKAISDARLKTLDIHYNESKPTTIQNTGKLVRINFKGGYISGGFLPNE   60
ADK39130.1          MPQQLSPINIETKKAISDTRLKTLDIHYNESKPTTIQNTGKLVRINFKGGYISGGFLPNE   60
NP_536532.1         MPQQLSPINIETKKAISDTRLKTLDIHYNESKPTTIQNTGKLVRINFKGGYISGGFLPNE   60
QNI38872.1          MPQQLSPINIETKKAISDTRLKTLDIHYNESKPTTIQNTGKLVRINFKGGYISGGFLPNE   60
                    ******************:*****************************************

CAA66449.1          YVLSTIHIYWGKEDDYGSNHLIDVYKYSGEINLVHWNKKKYSSYEEAKKHDDGIIIIAIF   120
WDQ84781.1          YVLSTIHIYWGKEDDYGSNHLIDVYKYSGEINLVHWNKKKYSSYEEAKKHDDGIIIIAIF   120
WBT04084.1          YVLSTIHIYWGKEDDYGSNHLIDVYKYSGEINLVHWNKKKYSSYEEAKKHDDGIIIIAIF   120
UYW97776.1          YVLSTIHIYWGKEDDYGSNHLIDVYKYSGEINLVHWNKKKYSSYEEAKKHDDGIIIIAIF   120
WFD73904.1          YVLSTIHIYWGKEDDYGSNHLIDVYKYSGEINLVHWNKKKYSSYEEAKKHDDGIIIIAIF   120
WDO63050.1          YVLSTIHIYWGKEDDYGSNHLIDVYKYSGEINLVHWNKKKYSSYEEAKKHDDGIIIIAIF   120
UZT62701.1          YVLSTIHIYWGKEDDYGSNHLIDVYKYSGEINLVHWNKKKYSSYEEAKKHDDGIIIIAIF   120
UZV08183.1          YVLSTIHIYWGKEDDYGSNHLIDVYKYSGEINLVHWNKKKYSSYEEAKKHDDGIIIIAIF   120
AAU01309.1          YVLSTIHIYWGKEDDYGSNHLIDVYKYSGEINLVHWNKKKYSSYEEAKKHDDGIIIIAIF   120
AUW64186.1          YVLSTIHIYWGKEDDYGSNHLIDVYKYSGEINLVHWNKKKYSSYEEAKKHDDGIIIIAIF   120
WFJ79428.1          YVLSTIHIYWGKEDDYGSNHLIDVYKYSGEINLVHWNKKKYSSYEEAKKHNDGIIIIAIF   120
WCC70104.1          YVLSTIHIYWGKEDDYGSNHLIDVYKYSGEINLVHWNKKKYSSYEEAKKHDDGIIIIAIF   120
UYD49617.1          YVLSTIHIYWGKEDDYGSNHLIDVYKYSGEINLVHWNKKKYSSYEEAKKHDDGIIIIAIF   120
AAY97104.1          YVLSTIHIYWGKEDDYGSNHLIDVYKYSGEINLVHWNKKKYSSYEEAKKHDDGIIIIAIF   120
YP_010377102.1      YVLSTIHIYWGKEDDYGSNHLIDVYKYSGEINLVHWNKKKYSSYEEAKKHDDGIIIIAIF   120
ADK39130.1          YVLSTIHIYWGKEDDYGSNHLIDVYKYSGEINLVHWNKKKYSSYEEAKKHDDGIIIIAIF   120
NP_536532.1         YVLSTIHIYWGKEDDYGSNHLIDVYKYSGEINLVHWNKKKYSSYEEAKKHDDGIIIIAIF   120
QNI38872.1          YVLSTIHIYWGKEDDYGSNHLIDVYKYSGEINLVHWNKKKYSSYEEAKKHDDGIIIIAIF   120
                    **************************************************:*********

CAA66449.1          LQVSDHKNVYFQKIVNQLDSIRSANMSAPFDSVFYLDNLLPSTLDYFTYLGTTINHSADA   180
WDQ84781.1          LQVSDHKNVYFQKIVNQLDSIRSANMSAPFDSVFYLDNLLPSTLDYFTYLGTTINHSADA   180
WBT04084.1          LQVSDHKNVYFQKIVNQLDSIRSANMSAPFDSVFYLDNLLPSTLDYFTYLGTTINHSADA   180
UYW97776.1          LQVSDHKNVYFQKIVNQLDSIRSANMSAPFDLVFYLDNLLPSTLDYFTYLGTTINHSADA   180
WFD73904.1          LQVSDHKNVYFQKIVNQLDSIRSANMSAPFDSVFYLDNLLPSTLDYFTYLGTTINHSADA   180
WDO63050.1          LQVSDHKNVYFQKIVNQLDSIRSANMSAPFDSVFYLDNLLPSTLDYFTYLGTTINHSADA   180
UZT62701.1          LQVSDHKNVYFQKIVNQLDSIRSANMSAPFDSVFYLDNLLPSTLDYFTYLGTTINHSADA   180
UZV08183.1          LQVSDHKNVYFQKIVNQLDSIRSANMSAPFDSVFYLDNLLPSTLDYFTYLGTTINHSADA   180
AAU01309.1          LQVSDHKNVYFQKIVNQLDSIRSANMSAPFDSVFYLDNLLPSTLDYFTYLGTTINHSADA   180
AUW64186.1          LQVSDHKNVYFQKIVNQLDSIRSANMSAPFDSVFYLDNLLPSTLDYFTYLGTTINHSADA   180
WFJ79428.1          LQVSDHKNVYFQKIVNQLDSIRSANMSAPFDSVFYLDNLLPSTLDYFTYLGTTINHSADA   180
WCC70104.1          LQVSDHKNVYFQKIVNQLDSIRSANMSAPFDSVFYLDNLLPSTLDYFTYLGTTINHSADA   180
UYD49617.1          LQVSDHKNVYFQKIVNQLNSIRSANMSAPFDSVFYLDNLLPSTLDYFTYLGTTINHSADA   180
AAY97104.1          LQVSDHKNVYFQKIVNQLDSIRSANMSAPFDSVFYLDNLLPSTLDYFTYLGTTINHSADA   180
YP_010377102.1      LQVSDHKNVYFQKIVNQLDSIRSANMSAPFDSVFYLDNLLPSTLDYFTYLGTTINHSADA   180
ADK39130.1          LQVSDHKNVYFQKIVNQLDSIRSANMSAPFDSVFYLDNLLPSTLDYFTYLGTTINHSADA   180
NP_536532.1         LQVSDHKNVYFQKIVNQLDSIRSANMSAPFDSVFYLDNLLPSTLDYFTYLGTTINHSADA   180
QNI38872.1          LQVSDHKNVYFQKIVNQLDSIRSANMSAPFDSVFYLDNLLPSTLDYFTYLGTTINHSADA   180
                    ******************:************ ****************************
```

```
CAA66449.1          VWIIFPTPINIHSDQLSKFRTLLSSSNHEGKPHYITENYRNPYKLNDDTQVYYSGEIIRA     240
WDQ84781.1          AWIIFPTPINIHFDQLSKFRTLLSSSNHEGKPHYITENYRNPYKLNDDTQVYYSGEIIRA     240
WBT04084.1          AWIIFPTPINIHSDQLSKFRTLLSSSNHEGKPHYITENYRNSYKLNDDTQVYYSGEIIRA     240
UYW97776.1          AWIIFPTPINIHSDQLSKFRTLLSSSNHEGKPHYITENYRNPYKLNDDTQVYYSGEIIRA     240
WFD73904.1          AWIIFPTPINIHSDQLSKFRTLLSSSNHEGKPHYITENYRNPYKLNDDTQVYYSGEIIRA     240
WDO63050.1          AWIIFPTPINIHSDQLSKFRTLLSSSNHEGKPHYITENYRNPYKLNDDTQVYYSGEIIRA     240
UZT62701.1          AWIIFPTPINIHSDQLSKFRTLLSSSNHEGKPHYITENYRNPYKLNDDTQVYYSGEIIRA     240
UZV08183.1          AWIIFPTPINIHSDQLSKFRTLLSSSNHEGKPHYITENYRNPYKLNDDTQVYYSGEIIRA     240
AAU01309.1          AWIIFPTPINIHSDQLSKFRTLLSSSNHEGKPHYITENYRNPYKLNDDTQVYYSGEIIRA     240
AUW64186.1          AWIIFPTPINIHSDQLSKFRTLLSSSNHEGKPHYITENYRNPYKLNDDTQVYYSGEIIRA     240
WFJ79428.1          AWIIFPTPINIHSDQLSKFRTLLSSSNHEGKPHYITENYRNPYKLNDDTQVYYSGEIIRA     240
WCC70104.1          AWIIFPTPINIHSDQLSKFRTLLSSSNHEGKPHYITENYRNPYKLNDDTQVYYSGEIIRA     240
UYD49617.1          AWIIFPTPINIHSDQLSKFRTLLSSSNHEGKPHYITENYRNPYKLNDDTQVYYSGEIIRA     240
AAY97104.1          AWIIFPTPINIHSDQLSKFRTLLSSSNHEGKPRYITENYRNPYKLNDDTQVYYSGEIIRA     240
YP_010377102.1      AWIIFPTPINIHSDQLSKFRTLLSSSNHEGKPHYITENYRNPYKLNDDTQVYYSGEIIRA     240
ADK39130.1          AWIIFPTPINIHSDQLSKFRTLLSSSNHEGKPHYITENYRNPYKLNDDTQVYYSGEIIRA     240
NP_536532.1         AWIIFPTPINIHSDQLSKFRTLLSSSNHEGKPHYITENYRNPYKLNDDTQVYYSGEIIRA     240
QNI38872.1          AWIIFPTPINIHSDQLSKFRTLLSSSNHEGKPHYITENYRNPYKLNDDTQVYYSGEIIRA     240
                    .*********** *******************:******** ******************

CAA66449.1          ATTSPVRENYFMKWLSDLREVCFSYYQKYIEGNKTFAIIAIVFVFILTAILFLMSRQYSR     300
WDQ84781.1          ATTSPVRENYFMKWLSDLREACFSYYQKYIEGNKTFAIIAIVFVFILTAILFLMSQRYSR     300
WBT04084.1          ATTSPVRENYFMKWLSDLREACFSYYQKYIEGNKTFAIIAIVFVFILTAILFLMSQRYSR     300
UYW97776.1          ATTSPVRENYFMKWLSDLREACFSYYQKYIEGNKTFAIIAIVFVFILTAILFLMSQRYSR     300
WFD73904.1          ATTLPVRENYFMKWLSDLREACFSYYQKYIEGNKTFAIIAIVFVFILTAILFLMSQRYSR     300
WDO63050.1          ATTSPVRENYFMKWLSDLREACFSYYQKYIEGNKTFAIIAIVFVFILTAILFLMSQRYLR     300
UZT62701.1          ATTSPVRENYFMKWLSDLREACFSYYQKYIEGNKTFAIIAIVFVFILTAILFLISQRYSR     300
UZV08183.1          VTTSPVRENYFMKWLSDLREACFSYYQKYIEGNKTFAIIAIVFVFILTAILFLMSQRYSR     300
AAU01309.1          ATTSPVRENYFMKWLSDLREVCFSYYQKYIEGNKTFAIIAIVFVFILTAILFLMSQRYSR     300
AUW64186.1          ATTSPVRENYFMKWLSDLRKACFSYYQKYIEGNKTFAIIAIVFVFILTAILFLMSQRYSR     300
WFJ79428.1          ATTSPVRENYFMKWLSDLREACFSYYQKYIEGNKTFAIIAIVFVFILTAILFLMSQRYSR     300
WCC70104.1          ATTSPVRENYFMKWLSDLREACFSYYQKYIKGNKTFAIIAIVFVFILTAILFLMSQRYSR     300
UYD49617.1          ATTSPVRENYFMKWLSDLREACFSYYQKYIEGNKTFAIIAIVFVFILTAILFLMSQRYSR     300
AAY97104.1          ATTSPVRENYFMKWLSDLREACFSYYQKYIEGNKTFAIIAIVFVFILTAILFLMSQRYSR     300
YP_010377102.1      ATTSPVRENYFMKWLSDLREACFSYYQKYIEGNKTFAIIAIVFVFILTAILFLMSQRYSR     300
ADK39130.1          ATTSPVRENYFMKWLSDLREACFSYYQKYIEGNKTFAIIAIVFVFILTTILFLMSQRYSR     300
NP_536532.1         ATTSPVRENYFMKWLSDLREACFSYYQKYIEGNKTFAIIAIVFVFILTAILFLMSQRYSR     300
QNI38872.1          ATTSPMRENYFMKWLSDLREACFSYYQKYIEGNKTFAIIAIVFVFILTAILFLMSQRYSR     300
                    .** *:*************:.*********:*****************:****:*::* *

CAA66449.1          EKQN       304
WDQ84781.1          EKQN       304
WBT04084.1          EKQN       304
UYW97776.1          EKQN       304
WFD73904.1          EKQN       304
WDO63050.1          EKQN       304
UZT62701.1          EKQN       304
UZV08183.1          EKQN       304
AAU01309.1          EKQN       304
AUW64186.1          EKQN       304
WFJ79428.1          EKQN       304
WCC70104.1          EKQN       304
UYD49617.1          EKQN       304
AAY97104.1          EKQN       304
YP_010377102.1      EKQN       304
ADK39130.1          EKQN       304
NP_536532.1         EKQN       304
QNI38872.1          EKQN       304
                    ****
```
