# Supplementary material for: In silico design and immunoinformatics analysis of a universal multi-epitope vaccine against monkeypox virus
Source: PLoS One. 2023 May 23;18(5):e0286224. doi: 10.1371/journal.pone.0286224 (PMC10205007; doi:10.1371/journal.pone.0286224)
Supplement: S2 Data — (PDF) [file pone.0286224.s002.pdf]

Multiple sequence alignment of envelope protein A28 homolog.

```
AAY97343.1      MNSLSIFFIVVATAAVCLLFIQSYSIYENYGNIKEFNATHAAFEYSKSIGGTPALDRRVQ     60
AAY96942.1      MNSLSIFFIVVATAAVCLLFIQSYSIYENYGNIKEFNATHAAFEYSKSIGGTPALDRRVQ     60
UXK31194.1      MNSLSIFFIVVATAAVCLLFIQSYSIYENYGNIKEFNATHAAFEYSKSIGGTPALDRRVQ     60
WCZ63551.1      MNSLSIFFIVVATAAVCLLFIQSYSIYENYGNIKEFNATHAAFEYSKSIGGTPALDRRVQ     60
WCS91476.1      MNSLSIFFIVVATAAVCLLFIQSYSIYENYGNIKEFNATHAAFEYSKSIGGTPALDRRVQ     60
NP_536567.1     MNSLSIFFIVVATAAVCLLFIQSYSIYENYGNIKEFNATHAAFEYSKSIGGTPALDRRVQ     60
QNI40000.1      MNSLSIFFIVVATAAVCLLFIQSYSIYENYGNIKEFNATHAAFEYSKSIGGTPALDRRVQ     60
                ************************************************************

AAY97343.1      DVNDTISDVKQKWRCVVYPGNGFVSASIFGFQAEVGPNNTISIRKFNTMRQCIDFTFSDV     120
AAY96942.1      DVNDIISDVKQKWRCVVYPGNGFVSASIFGFQAEVGPNNTRSIRKFNTMRQCIDFTFSDV     120
UXK31194.1      DVNDTISDVKQKWRCVVYPGNGFVSASIFGFQAEVGPNNTRSIRKFNTMRQCIDFTFSNV     120
WCZ63551.1      DVNDTISDVKQKWRCVVYPGNGFVSASIFGFQAEVGPNNTRSIRKFNTMQQCIDFTFSDV     120
WCS91476.1      DVNDTISDVKQKWRCVVYPGNGFVSASIFGFQAKVGPNNTRSIRKFNTMRQCIDFTFSDV     120
NP_536567.1     DVNDTISDVKQKWRCVVYPGNGFVSASIFGFQAEVGPNNTRSIRKFNTMRQCIDFTFSDV     120
QNI40000.1      DVNDTISDVKQKWRCVVYPGNGFVSASIFGFQAEVGPNNSRSIRKFNTMRQCIDFTFSDV     120
                **** ****************************:*****: ********:********:*

AAY97343.1      ININDIYNPCIAPNINNTECQFLKSVL          146
AAY96942.1      ININDIYNPCIAPNINNTECQFLKSVL          146
UXK31194.1      ININDIYNPCIAPNINNTECQFLKSVL          146
WCZ63551.1      ININDIYNPCIAPNINNTECQFLKSVL          146
WCS91476.1      ININDIYNPCIAPNINNTECQFLKSVL          146
NP_536567.1     ININDIYNPCIAPNINNTECQFLKSVL          146
QNI40000.1      ININDIYNPCIAPNINNTECQFLKSVL          146
                **************************
```
