# Supplementary material for: In silico design and immunoinformatics analysis of a universal multi-epitope vaccine against monkeypox virus
Source: PLoS One. 2023 May 23;18(5):e0286224. doi: 10.1371/journal.pone.0286224 (PMC10205007; doi:10.1371/journal.pone.0286224)
Supplement: S1 Table — (DOCX) [file pone.0286224.s003.docx]

**Table S1:** The predicted CTL epitopes from the cell surface-binding protein.

| **Epitope** | **Allele** | **Antigenicity score** | **Allergenicity** | **Toxicity** | **Present in conserved regions** |
| --- | --- | --- | --- | --- | --- |
| APFDSVFYL | HLA-A*0201, HLA-B*3501, HLA-B*3901, HLA-B*5101, HLA-B*5102, HLA-B*5301, HLA-B7, HLA-Cw*0401 | 0.2814 | Allergen | Non-toxin | No |
| DQLSKFRTL | HLA-B*3902, HLA-B14, HLA-B8, HLA-Cw*0301 | -0.7588 | Non-allergen | Non-toxin | Yes |
| **FAIIAIVFV** | **HLA-B*51, HLA-B*5101, HLA-B*5102, HLA-B*5103, HLA-B*5401** | **1.3444** | **Non-allergen** | **Non-toxin** | **Yes** |
| GEIIRAATT | HLA-B*4403, HLA-B40, HLA-B61 | -0.2355 | Allergen | Non-toxin | No |
| QLSKFRTLL | HLA-A*0201, HLA-A2, HLA-A2.1 | -0.8785 | Non-allergen | Non-toxin | Yes |
| RENYFMKWL | HLA-B40, HLA-B60, HLA-B61, HLA-Cw*0602 | 0.0580 | Non-allergen | Non-toxin | Yes |
| **RLKTLDIHY** | **HLA-B*0702, HLA-B*3501, HLA-B62** | **1.9035** | **Non-allergen** | **Non-toxin** | **Yes** |
| SPVRENYFM | HLA-B*3501, HLA-B*5301, HLA-B7 | 0.7182 | Non-allergen | Non-toxin | No |
| SVFYLDNLL | HLA-A*0205, HLA-B7, HLA-Cw*0602 | 0.0793 | Non-allergen | Non-toxin | No |
| VRINFKGGY | HLA-B*2702, HLA-B*2705, HLA-Cw*0702 | 2.2055 | Allergen | Non-toxin | Yes |
| YFQKIVNQL | HLA-A24, HLA-Cw*0401, HLA-Cw*0602 | -0.5633 | Allergen | Non-toxin | Yes |
| YKYSGEINL | HLA-B*3902,HLA-B*51, HLA-B*5301, HLA-B*5401, HLA-B14 | 0.4526 | Allergen | Non-toxin | Yes |

The selected epitopes have been shown in bold.
