# Supplementary material for: In silico design and immunoinformatics analysis of a universal multi-epitope vaccine against monkeypox virus
Source: PLoS One. 2023 May 23;18(5):e0286224. doi: 10.1371/journal.pone.0286224 (PMC10205007; doi:10.1371/journal.pone.0286224)
Supplement: S2 Table — (DOCX) [file pone.0286224.s004.docx]

**Table S2:** The predicted CTL epitopes from the envelope protein A28 homolog.

| **Epitope** | **Allele** | **Antigenicity score** | **Allergenicity** | **Toxicity** | **Present in conserved regions** |
| --- | --- | --- | --- | --- | --- |
| AAFEYSKSI | HLA-B*5101, HLA-B*5102, HLA-B*5103 | 0.3937 | Allergen | Non-toxin | Yes |
| **ALDRRVQDV** | **HLA-A2, HLA-A*0201, HLA-A2.1, HLA-B14, HLA-B*3901** | **1.117** | **Non-allergen** | **Non-toxin** | **Yes** |
| ECQFLKSVL | HLA-B*3902, HLA-B60, HLA-B8 | -0.4521 | Allergen | Non-toxin | Yes |
| **FTFSDVINI** | **HLA-B*5301, HLA-B*5401, HLA-B*51** | **1.0007** | **Non-allergen** | **Non-toxin** | **Yes** |
| **ISDVKQKWR** | **HLA-A1, HLA-A*3302, HLA-A68.1** | **1.6356** | **Non-allergen** | **Non-toxin** | **Yes** |
| **KEFNATHAA** | **HLA-B40, HLA-B*4403, HLA-B61, HLA-B*0702** | **1.079** | **Non-allergen** | **Non-toxin** | **Yes** |
| KQKWRCVVY | HLA-B*2702, HLA-B*2705, HLA-B*3501  HLA-B62, HLA-B*0702 | 1.7426 | Allergen | Non-toxin | Yes |
| KSIGGTPAL | HLA-A24, HLA-B14, HLA-B*3501, HLA-B*3801, HLA-B*3901  HLA-B*3902, HLA-B60  HLA-B7, HLA-B*0702  HLA-Cw*0301 | 0.2666 | Non-allergen | Non-toxin | Yes |
| QSYSIYENY | HLA-B*3501, HLA-B*5801, HLA-Cw*0702 | 0.3961 | Non-allergen | Non-toxin | Yes |
| RRVQDVNDT | HLA-B14, HLA-B*2702, HLA-B*2705 | 1.3228 | Allergen | Non-toxin | No |
| RSIRKFNTM | HLA-B*3501, HLA-B*5801, HLA-Cw*0301 | -0.2927 | Non-allergen | Non-toxin | No |
| SDVINIDIY | HLA-B*3701, HLA-B*4403, HLA-Cw*0702 | 0.1779 | Allergen | Non-toxin | Yes |
| **SIFGFQAEV** | **HLA-A2, HLA-A*0201, HLA-A*0205** | **0.4227** | **Non-allergen** | **Non-toxin** | **Yes** |
| SIRKFNTMR | HLA-A3, HLA-A*3101, HLA-A*3302 | -1.1081 | Allergen | Non-toxin | Yes |
| SIYENYGNI | HLA-A*0205, HLA-B*3901, HLA-Cw*0301 | 0.3145 | Non-allergen | Non-toxin | Yes |
| **SLSIFFIVV** | **HLA-A2, HLA-A*0201**  **HLA-A3, HLA-A2.1, HLA-B62** | **0.8068** | **Non-allergen** | **Non-toxin** | **Yes** |
| TECQFLKSV | HLA-B40, HLA-B*4403, HLA-B60, HLA-B61, HLA-Cw*0602 | -0.0525 | Allergen | Non-toxin | Yes |
| VATAAVCLL | HLA-B*3901, HLA-B*3902, HLA-B*5101  HLA-B*5102, HLA-B*5103, HLA-B60, HLA-B7, HLA-B8, HLA-Cw*0602 | 0.3136 | Non-allergen | Non-toxin | Yes |
| VGPNNTRSI | HLA-B*5101, HLA-B*5102, HLA-B*5103 | 1.1958 | Non-allergen | Non-toxin | No |
| **VVATAAVCL** | **HLA-A*0205, HLA-B*3801, HLA-B*3902, HLA-B7** | **0.5781** | **Non-allergen** | **Non-toxin** | **Yes** |
| YPGNGFVSA | HLA-B*5301, HLA-B*5401, HLA-B*51 | -0.3968 | Non-allergen | Non-toxin | Yes |

The selected epitopes have been shown in bold.
