# Supplementary material for: In silico design and immunoinformatics analysis of a universal multi-epitope vaccine against monkeypox virus
Source: PLoS One. 2023 May 23;18(5):e0286224. doi: 10.1371/journal.pone.0286224 (PMC10205007; doi:10.1371/journal.pone.0286224)
Supplement: S3 Table — (DOCX) [file pone.0286224.s005.docx]

**Table S3:** The predicted HTL epitopes from the cell surface-binding protein.

| **Epitop** | **Allele** | **Antigenicity score** | **Allergenicity** | **Toxicity** | **Present in conserved regions** | **IFN-γ –inducing** | **IL4- inducing** |
| --- | --- | --- | --- | --- | --- | --- | --- |
| FILTAILFL | DRB1_0701, DRB1_0703, DRB1_1502, DRB1_1101, DRB1_0421, DRB1_1128, DRB1_1305 | 0.5392 | Non-allergen | Non-toxin | Yes | Positive | Non-inducer |
| FLMSQRYSR | DRB1_0309, DRB5_0101, DRB5_0105, DRB1_1120, DRB1_1302 | 0.9843 | Non-allergen | Non-toxin | No | Positive | Inducer |
| FRTLLSSSN | DRB1_0405, DRB1_0408, DRB1_0101, DRB1_0410 | -0.274 | Non-allergen | Non-toxin | Yes | Positive | Non-inducer |
| **FSYYQKYIE** | **DRB1_0801, DRB1_0817, DRB1_0806** | **0.6113** | **Non-allergen** | **Non-toxin** | **Yes** | **Positive** | **Inducer** |
| FYLDNLLPS | DRB1_0305, DRB1_0306, DRB1_0307, DRB1_0308, DRB1_0309, DRB1_0311, DRB1_0301, DRB1_0401, DRB1_0426, DRB1_0421, DRB1_1107 | 0.2071 | Non-allergen | Non-toxin | Yes | Positive | Non-inducer |
| IIIIAIFLQ | DRB1_0404, | 0.3862 | Non-allergen | Non-toxin | Yes | Positive | Non-inducer |
|  | DRB1_0423, DRB1_1107, DRB1_1304, DRB1_0410, DRB1_1102, DRB1_1104, DRB1_1106, DRB1_1114, DRB1_1121, DRB1_1307, DRB1_1311, DRB1_1322, DRB1_1323, DRB1_0305, DRB1_0402, DRB1_0804, DRB1_0806, DRB1_1101, DRB1_0301, DRB1_0306, DRB1_0307, DRB1_0308, DRB1_0311, DRB1_0408, DRB1_0813, DRB1_0802, DRB1_1321, DRB5_0101, DRB5_0105 |  |  |  |  |  |  |
| INIETKKAI | DRB1_1301, DRB1_1327, DRB1_1328 | 0.9183 | Allergen | Non-toxin | Yes | Positive | Inducer |
| IRAATTSPV | DRB1_0404, DRB1_0423, DRB1_0410, DRB1_0408 | 0.4958 | Allergen | Non-toxin | No | Positive | Inducer |
| LFLMSQRYS | DRB1_1102, DRB1_1114, DRB1_1121, DRB1_1322, DRB1_1323, DRB1_1301, DRB1_1327, DRB1_1328, DRB1_1120, DRB1_1302, DRB1_0402, DRB1_0804, DRB1_1304, DRB1_1307 | 0.9976 | Allergen | Non-toxin | No | Positive | Inducer |
| LSKFRTLLS | DRB1_0402, DRB1_0404, DRB1_0423 | -0.735 | Non-allergen | Non-toxin | Yes | Positive | Inducer |
| LVHWNKKKY | DRB1_1301, DRB1_1327, DRB1_1328, DRB1_1120, DRB1_1302, DRB1_1304, DRB1_1102, DRB1_1121, DRB1_1322, DRB1_1114, DRB1_1323 | 1.041 | Allergen | Non-toxin | Yes | Positive | Inducer |
| VFILTAILF | DRB1_0102, DRB1_0101, DRB5_0101, DRB5_0105, DRB1_1128, DRB1_1305, DRB1_1104, DRB1_1106, DRB1_1311, DRB1_1501, DRB1_1506 | 0.3203 | Non-allergen | Non-toxin | Yes | Positive | Non-inducer |
| **VHWNKKKYS** | **DRB1_1301, DRB1_1327, DRB1_1328** | **1.138** | **Non-allergen** | **Non-toxin** | **Yes** | **Positive** | **Inducer** |
| VRINFKGGY | DRB1_0301, DRB1_0806, DRB1_0309, DRB1_0804, DRB1_1107, DRB1_0802, DRB1_1104, DRB1_1106, DRB1_1301, DRB1_1311, DRB1_1327, DRB1_1328, DRB1_0305, DRB1_0801, DRB1_1128, DRB1_1305, DRB1_1307, DRB1_1101, DRB1_1304 | 2.2055 | Allergen | Non-toxin | Yes | Positive | Inducer |
| VYFQKIVNQ | DRB1_1102, DRB1_1121, DRB1_1304, DRB1_1322, DRB1_1114, DRB1_1323, DRB1_0306, DRB1_0307, DRB1_0308, DRB1_0311 | -0.063 | Non-allergen | Non-toxin | Yes | Positive | Inducer |
| WIIFPTPIN | DRB1_0405, DRB1_0408, DRB1_0817, DRB1_1502, DRB1_1120, DRB1_1302, DRB1_1321, DRB1_0410 | 0.2427 | Allergen | Non-toxin | Yes | Positive | Inducer |
| WLSDLREAC | DRB1_0305, DRB1_0306, DRB1_0307, DRB1_0308, DRB1_0311, DRB1_0309, DRB1_1107 | 0.5041 | Allergen | Non-toxin | No | Positive | Inducer |
| YFMKWLSDL | DRB1_0813, DRB1_0802, DRB1_0801 | -0.835 | Non-allergen | Non-toxin | Yes | Positive | Non-inducer |
| YRNPYKLND | DRB1_0817, DRB1_1321, DRB1_0801 | 0.451 | Allergen | Non-toxin | No | Positive | Inducer |
| **YVLSTIHIY** | **DRB1_1120, DRB1_1302, DRB1_0421, DRB1_1114, DRB1_1323, DRB5_0101, DRB5_0105, DRB1_0401, DRB1_0426, DRB1_0701,**  **DRB1_0703** | **0.5976** | **Non-allergen** | **Non-toxin** | **Yes** | **Positive** | **Inducer** |

The selected epitopes have been shown in bold.
