# Supplementary material for: In silico design and immunoinformatics analysis of a universal multi-epitope vaccine against monkeypox virus
Source: PLoS One. 2023 May 23;18(5):e0286224. doi: 10.1371/journal.pone.0286224 (PMC10205007; doi:10.1371/journal.pone.0286224)
Supplement: S4 Table — (DOCX) [file pone.0286224.s006.docx]

**Table S4:** The predicted HTL epitopes from the envelope protein A28 homolog.

| **Epitope** | **Allele** | **Antigenicity score** | **Allergenicity** | **Toxicity** | **Present in conserved regions** | **IFN-γ –inducing** | **IL4- inducing** |
| --- | --- | --- | --- | --- | --- | --- | --- |
| FFIVVATAA | DRB1_0101, DRB1_0813, DRB5_0101, DRB5_0105, DRB1_0102, DRB1_0405, DRB1_0408, DRB1_0305 | 0.7638 | Non-allergen | Non-toxin | Yes | Positive | Non-inducer |
| FIVVATAAV | DRB1_0405, DRB1_0408, DRB1_0309, DRB1_0421, DRB1_1101, DRB1_1128, DRB1_1305, DRB1_1307, DRB1_0401, DRB1_0426, DRB1_0802, DRB1_0404, DRB1_0410, DRB1_0423, DRB1_0813, DRB1_1321 | 0.6997 | Non-allergen | Non-toxin | Yes | Positive | Non-inducer |
| **FTFSDVINI** | **DRB1_0305, DRB1_0701, DRB1_0703, DRB1_0309, DRB1_0421, DRB1_0401, DRB1_0426, DRB1_1101, DRB1_0306, DRB1_0307, DRB1_0308, DRB1_0311, DRB1_1128, DRB1_1305** | **1.0007** | **Non-allergen** | **Non-toxin** | **Yes** | **Positive** | **Inducer** |
| FVSASIFGF | DRB1_0301, DRB1_0309, DRB1_1120, DRB1_1128, DRB1_1302, DRB1_1305, DRB1_1114, DRB1_1301, DRB1_1323, DRB1_1327, DRB1_1328, DRB1_0305, DRB1_1321, DRB5_0101, DRB5_0105, DRB1_0701, DRB1_0703, DRB1_1307, DRB1_1502 | 0.3511 | Non-allergen | Non-toxin | Yes | Positive | Inducer |
| IDIYNPCIA | DRB1_0804, DRB1_0802, DRB1_0806 | 0.3287 | Allergen | Non-toxin | Yes | Positive | Inducer |
| IFFIVVATA | DRB1_1307, DRB1_0301, DRB1_0306, DRB1_0307, DRB1_0308, DRB1_0311, DRB1_0401, DRB1_0404, DRB1_0410, DRB1_0423, DRB1_0426, DRB1_1101, DRB1_1104, DRB1_1106, DRB1_1107, DRB1_1311, DRB1_1321, DRB1_0802, DRB1_0804, DRB1_0305, DRB1_0408, DRB1_0806, DRB1_0813, DRB1_1128, DRB1_1305, DRB1_0309, DRB1_0402 | 0.8374 | Non-allergen | Non-toxin | Yes | Positive | Non-inducer |
| INIDIYNPC | DRB1_0306, DRB1_0307, DRB1_0308, DRB1_0311, DRB1_0301, DRB1_1107, DRB1_0401, DRB1_0426 | -0.0445 | Non-allergen | Non-toxin | Yes | Positive | Inducer |
| IQSYSIYEN | DRB1_1304, DRB1_0806, DRB1_0817, DRB1_0801 | 0.0543 | Non-allergen | Non-toxin | Yes | Positive | Inducer |
| IRKFNTMRQ | DRB1_0401, DRB1_0402, DRB1_0404, DRB1_0405, DRB1_0408, DRB1_0410, DRB1_0423, DRB1_0426, DRB1_0801, DRB1_0806, DRB1_0813, DRB1_0817, DRB1_1101, DRB1_1102, DRB1_1104, DRB1_1106, DRB1_1107, DRB1_1114, DRB1_1121, DRB1_1301, DRB1_1304, DRB1_1311, DRB1_1321, DRB1_1322, DRB1_1323, DRB1_1327, DRB1_1328, DRB1_1501, DRB1_1506, DRB1_0305, DRB1_0802, DRB1_0804, DRB1_1120, DRB1_1128, DRB1_1302, DRB1_1305, DRB1_1307, DRB1_1502, DRB1_0306, DRB1_0307, DRB1_0308, DRB1_0311, DRB1_0301, DRB1_0421 | -1.2490 | Allergen | Non-toxin | Yes | Positive | Inducer |
| IVVATAAVC | DRB1_0102, DRB1_1102, DRB1_1104, DRB1_1106, DRB1_1121, DRB1_1311, DRB1_1322, DRB1_0101, DRB1_0306, DRB1_0307, DRB1_0308, DRB1_0311, DRB1_0402, DRB1_0404, DRB1_0423, DRB1_1107, DRB1_1114, DRB1_1307, DRB1_1323, DRB1_0804, DRB1_1101 | 0.3273 | Non-allergen | Non-toxin | Yes | Positive | Non-inducer |
| LSIFFIVVA | DRB1_1102, DRB1_1121, DRB1_1322, DRB1_1114, DRB1_1323, DRB1_1501, DRB1_1506, DRB1_1304 | 0.8665 | Non-allergen | Non-toxin | Yes | Positive | Non-inducer |
| MNSLSIFFI | DRB1_1104, DRB1_1106, DRB1_1120, DRB1_1301, DRB1_1302, DRB1_1304, DRB1_1311, DRB1_1327, DRB1_1328, DRB1_1501, DRB1_1506, DRB1_0102, DRB1_0301, DRB1_1102, DRB1_1121, DRB1_1322 | 0.6559 | Non-allergen | Non-toxin | Yes | Positive | Non-inducer |
| VGPNNTRSI | DRB1_0402, DRB1_1120, DRB1_1301, DRB1_1302, DRB1_1304, DRB1_1327, DRB1_1328, DRB1_1102, DRB1_1121, DRB1_1322, DRB1_1114, DRB1_1323 | 1.1958 | Non-allergen | Non-toxin | No | Positive | Inducer |
| **VKQKWRCVV** | **DRB1_0802, DRB1_0804, DRB1_0801, DRB1_0806, DRB1_0813, DRB1_0817, DRB1_1120, DRB1_1301, DRB1_1302, DRB1_1327,** | **2.148** | **Non-allergen** | **Non-toxin** | **Yes** | **Positive** | **Inducer** |
|  | **DRB1_1328** |  |  |  |  |  |  |
| **VVATAAVCL** | **DRB1_0701, DRB1_0703, DRB1_0102, DRB1_0101** | **0.5781** | **Non-allergen** | **Non-toxin** | **Yes** | **Positive** | **Inducer** |
| YENYGNIKE | DRB1_0817, DRB1_0405, DRB1_1502, DRB1_0801 | 0.489 | Allergen | Non-toxin | Yes | Positive | Inducer |

The selected epitopes have been shown in bold.
