# Supplementary material for: In silico design and immunoinformatics analysis of a universal multi-epitope vaccine against monkeypox virus
Source: PLoS One. 2023 May 23;18(5):e0286224. doi: 10.1371/journal.pone.0286224 (PMC10205007; doi:10.1371/journal.pone.0286224)
Supplement: S5 Table — (DOCX) [file pone.0286224.s007.docx]

**Table S5:** The predicted LBL epitopes from the cell surface-binding protein.

| **Rank** | **Epitope** | **Start position** | **Score** | **Antigenicity score** | **Allergenicity** | **Toxicity** | **Present in conserved regions** |
| --- | --- | --- | --- | --- | --- | --- | --- |
| **1** | **CFSYYQKYIEGNKTFAIIAI** | **282** | **0.89** | **0.6454** | **Non-allergen** | **Non-toxin** | **Yes** |
| **2** | **HLIDVYKYSGEINLVHWNKK** | **100** | **0.87** | **0.6297** | **Non-allergen** | **Non-toxin** | **Yes** |
| 3 | AIFLQVSDHKNVYFQKIVNQ | 138 | 0.86 | 0.3169 | Allergen | Non-toxin | Yes |
| 3 | YVLSTIHIYWGKEDDYGSNH | 81 | 0.86 | 1.0342 | Allergen | Non-toxin | Yes |
| 4 | EDDYGSNHLIDVYKYSGEIN | 93 | 0.85 | -0.0024 | Allergen | Non-toxin | Yes |
| 4 | STLDYFTYLGTTINHSADAA | 182 | 0.85 | 0.5836 | Non-allergen | Non-toxin | No |
| 5 | KKYSSYEEAKKHDDGIIIIA | 119 | 0.83 | 0.4316 | Allergen | Non-toxin | Yes |
| **5** | **NVYFQKIVNQLDSIRSANMS** | **148** | **0.83** | **0.4289** | **Non-allergen** | **Non-toxin** | **Yes** |
| 5 | YKLNDDTQVYYSGEIIRAAT | 243 | 0.83 | -0.054 | Non-allergen | Non-toxin | No |
| 6 | AWIIFPTPINIHSDQLSKFR | 201 | 0.8 | -0.071 | Allergen | Non-toxin | No |
| **6** | **NLVHWNKKKYSSYEEAKKHD** | **112** | **0.8** | **0.7581** | **Non-allergen** | **Non-toxin** | **Yes** |
| 7 | LSDLREACFSYYQKYIEGNK | 275 | 0.79 | 0.6670 | Non-allergen | Non-toxin | No |
| 7 | TTSPVRENYFMKWLSDLREA | 262 | 0.79 | 0.1909 | Allergen | Non-toxin | No |
| 8 | SSSNHEGKPHYITENYRNPY | 224 | 0.76 | 0.5396 | Non-allergen | Non-toxin | No |
| 9 | SAPFDSVFYLDNLLPSTLDY | 167 | 0.75 | 0.4667 | Allergen | Non-toxin | No |
| 10 | LSPINIETKKAISDTRLKTL | 25 | 0.69 | 1.0733 | Allergen | Non-toxin | No |
| 11 | TGKLVRINFKGGYISGGFLP | 59 | 0.68 | 1.0377 | Allergen | Non-toxin | Yes |
| 12 | GTTINHSADAAWIIFPTPIN | 191 | 0.67 | 0.5517 | Allergen | Non-toxin | No |
| 13 | DQLSKFRTLLSSSNHEGKPH | 214 | 0.58 | -0.1560 | Non-allergen | Non-toxin | Yes |

The selected epitopes have been shown in bold.
