# Supplementary material for: In silico design and immunoinformatics analysis of a universal multi-epitope vaccine against monkeypox virus
Source: PLoS One. 2023 May 23;18(5):e0286224. doi: 10.1371/journal.pone.0286224 (PMC10205007; doi:10.1371/journal.pone.0286224)
Supplement: S6 Table — (DOCX) [file pone.0286224.s008.docx]

**Table S6:** The predicted LBL epitopes from the envelope protein A28 homolog.

| **Rank** | **Epitope** | **Start position** | **Score** | **Antigenicity score** | **Allergenicity** | **Toxicity** | **Present in conserved regions** |
| --- | --- | --- | --- | --- | --- | --- | --- |
| 1 | CVVYPGNGFVSASIFGFQAE | 75 | 0.89 | 0.1979 | Allergen | Non-toxin | Yes |
| 2 | VSASIFGFQAEVGPNNTRSI | 84 | 0.88 | 0.8242 | Allergen | Non-toxin | No |
| **3** | **FSDVINIDIYNPCIAPNINN** | **117** | **0.87** | **0.4474** | **Non-allergen** | **Non-toxin** | **Yes** |
| 4 | TISDVKQKWRCVVYPGNGFV | 65 | 0.86 | 0.8462 | Non-allergen | Non-toxin | No |
| 5 | QAEVGPNNTRSIRKFNTMRQ | 92 | 0.85 | 0.2418 | Non-allergen | Non-toxin | No |
| 5 | ALDRRVQDVNDTISDVKQKW | 54 | 0.85 | 1.0251 | Non-allergen | Non-toxin | No |
| 6 | QSYSIYENYGNIKEFNATHA | 22 | 0.84 | 0.5168 | Allergen | Non-toxin | Yes |
| 7 | TAAVCLLFIQSYSIYENYGN | 13 | 0.79 | 0.4419 | Non-allergen | Non-toxin | Yes |
| 7 | NATHAAFEYSKSIGGTPALD | 37 | 0.79 | 0.6142 | Non-allergen | Non-toxin | Yes |
| 8 | DIYNPCIAPNINNTECQFLK | 124 | 0.77 | 0.5575 | Non-allergen | Non-toxin | Yes |
| 9 | SKSIGGTPALDRRVQDVNDT | 46 | 0.75 | 0.7908 | Non-allergen | Non-toxin | No |
| 10 | NYGNIKEFNATHAAFEYSKS | 29 | 0.7 | 0.8109 | Allergen | Non-toxin | Yes |

The selected epitopes have been shown in bold.
