# Supplementary material for: In silico design and immunoinformatics analysis of a universal multi-epitope vaccine against monkeypox virus
Source: PLoS One. 2023 May 23;18(5):e0286224. doi: 10.1371/journal.pone.0286224 (PMC10205007; doi:10.1371/journal.pone.0286224)
Supplement: Attachment — Submitted filename: Response to Reviewers.docx [file pone.0286224.s009.docx]

**Manuscript No**: PONE-D-23-08627

**Title:** "*In silico* design and immunoinformatics analysis of a universal multi-epitope vaccine against monkeypox virus"

**Journal:** PLOS ONE

The authors of this manuscript thank the editor and reviewers for making suggestions and recommendations that helped us to improve the quality of the manuscript. We have made the changes suggested by the reviewers. All corrected sentences have been highlighted with Track Changes in the manuscript. We hope our revision has improved the paper to a level of their satisfaction. Number wise answers to their specific comments and suggestion are as follows.

**Journal Requirements:**

1. Please ensure that your manuscript meets PLOS ONE's style requirements, including those for file naming. The PLOS ONE style templates can be found at
   https://journals.plos.org/plosone/s/file?id=wjVg/PLOSOne_formatting_sample_main_body.pdf and 
   https://journals.plos.org/plosone/s/file?id=ba62/PLOSOne_formatting_sample_title_authors_affiliations.pdf

Ans: Thanks for the comments. All items were checked and corrected based on the PLOS ONE style templates.

2. We noticed you have some minor occurrence of overlapping text with the following previous publication(s), which needs to be addressed:

https://assets.cureus.com/uploads/review_article/pdf/102530/20220810-30968-1qo1gw.pdf
https://www.researchgate.net/publication/345757265_Monkeypox_Virus_in_Nigeria_Infection_Biology_Epidemiology_and_Evolution

In your revision ensure you cite all your sources (including your own works), and quote or rephrase any duplicated text outside the methods section. Further consideration is dependent on these concerns being addressed.

Ans: Thanks a lot for your attention. The previous publications were addressed (reference 5 in line # 49 and reference 14 in line #73).

3. Thank you for stating the following financial disclosure:
      "This study was financially supported by the Research Deputy of Shahrekord University of Medical Sciences with grant number 6411."

Please state what role the funders took in the study.  If the funders had no role, please state: "The funders had no role in study design, data collection and analysis, decision to publish, or preparation of the manuscript." 
If this statement is not correct you must amend it as needed. 
Please include this amended Role of Funder statement in your cover letter; we will change the online submission form on your behalf.

Ans: Dear editor, the above item was done accordingly.

4. Please note that funding information should not appear in any section or other areas of your manuscript. We will only publish funding information present in the Funding Statement section of the online submission form. Please remove any funding-related text from the manuscript.
     "Funding 
   This study was financially supported by the Research Deputy of Shahrekord University of Medical Sciences  with grant number 6411."

Ans: Dear editor, the above item was done accordingly.

5. PLOS requires an ORCID iD for the corresponding author in Editorial Manager on papers submitted after December 6th, 2016. Please ensure that you have an ORCID iD and that it is validated in Editorial Manager. To do this, go to ‘Update my Information’ (in the upper left-hand corner of the main menu), and click on the Fetch/Validate link next to the ORCID field. This will take you to the ORCID site and allow you to create a new iD or authenticate a pre-existing iD in Editorial Manager. Please see the following video for instructions on linking an ORCID iD to your Editorial Manager account: <https://www.youtube.com/watch?v=_xcclfuvtxQ>

Ans: Dear editor, the above item was done accordingly.

6. Your ethics statement should only appear in the Methods section of your manuscript. If your ethics statement is written in any section besides the Methods, please move it to the Methods section and delete it from any other section. Please ensure that your ethics statement is included in your manuscript, as the ethics statement entered into the online submission form will not be published alongside your manuscript.

Ans: It's done (line #268-270).

7. Please include captions for your Supporting Information files at the end of your manuscript, and update any in-text citations to match accordingly. Please see our Supporting Information guidelines for more information: http://journals.plos.org/plosone/s/supporting-information.

Ans: Thank you so much for your nice comment. It's done (line #962-978).

**Reviewer #1:**

1. The introduction section is weak in terms of referring to the environmental aspects of Monkeypox virus, https://doi.org/10.1515/reveh-2022-0221 should be used to strengthen this section.

Ans: As per your valuable comment , this has been included in the manuscript (line #58-59 and reference 9).

2. The authors chose the residues forming the disulfide bond based on a χ3 angle between -87° and +97° and an energy value less than 2.2 kcal/mol. Do you have a reference for these values? If you have a reference, be sure to give that cite.

Ans: It was done accordingly (line #378-380 and reference 66).

3. In section 2.2. give the reason for evaluating epitopes for inducing the production of IFN-γ and IL-4.

Ans: Thanks for the comments. It was done (line #156-158 and reference 47).

4. Accession numbers of TLR4 and cholera toxin B subunit should be mentioned in the MS.

Ans: Thanks a lot for the attention. It was done (line #170 and line #231).

5. Authors should explain why they used cholera toxin B subunit as an adjuvant by citing the source.

Ans: As per your valuable comment , this has been included in the manuscript (line #531-534 and reference 108-111).

**Reviewer #2:**

1. The abstract section is not well written. The abstract section needs more improvement with mentioning the specific finding (such as how may T-cell and B-cell epitopes etc.) were used for the vaccine construct instead of providing the overall findings.

Ans: Thank you so much for your nice comment. The abstract of the paper was thoroughly revised accordingly as the reviewer comment.

2. The main limitation of this study is the data size. The authors have used only 10 sequences for the cell surface-binding protein and only 5 sequences for the envelope protein A28 homolog. The authors could use more sequences because the authors have depended on the conserved regions of these proteins. There are 100 of sequences are available in the protein sequence database such as Uniprot or in NCBI protein database. It is not clear why authors used only limited numbers of sequences among the large number of sequences?

Ans: Thanks a lot for the attention. When we wrote this article, only these sequences were submitted and we only used them, according to your opinion, we also took the new sequences and analyzed them, and revised this part (line #120-124, line # 274-275).

3. Since this monkey pox virus is spreading in multiple countries, the analysis and showing the population coverage for the identified T-cell epitopes was important which is missing in this important study.

Ans: Thank you so much for your constructive comment. this has been included in the manuscript (line #173-178, line #310-318, line #545-546).

4. The authors could use 2 different servers for the allergenicity prediction for the cross validation of the allergenicity of the predicted epitopes.

Ans: Thank you for your valuable comment. If we use another server, we will lose some epitopes, and since the vaccine construct is designed, we cannot do this in this study, but we will definitely consider it in the next works. However, in many published articles, such as the articles below, one server has been used to determine the allergenicity of epitopes.

DOI: [10.3390/vaccines10050691](https://doi.org/10.3390/vaccines10050691)

DOI: [10.1038/s41598-022-12651-1](https://doi.org/10.1038/s41598-022-12651-1)

DOI: [10.3390/ijerph19095568](https://doi.org/10.3390/ijerph19095568)

DOI: [10.3390/vaccines10081300](https://doi.org/10.3390/vaccines10081300)

DOI: [10.1038/s41598-022-14427-z](https://doi.org/10.1038/s41598-022-14427-z)

DOI: [10.1038/s41598-023-30445-x](https://doi.org/10.1038/s41598-023-30445-x)

DOI: [10.1016/j.virusres.2020.198082](https://doi.org/10.1016/j.virusres.2020.198082)

5. The authors have mentioned about the in silico cloning in the materials and methods section, but they have not shown the Multiple cloning site in Figure 11 which needs correction and reanalysis of the in silico cloning for this vaccine construct.

Ans: This part and Figure 11 were revised based on the reviewer's comment, and the Multiple cloning site was marked in blue color in Figure 11 (line #257-266, line # 477-480).

6. The authors have predicted the three-dimensional structure of the final vaccine construct, so why did they predict the secondary structural features of the vaccine construct. Please provide a strong rational of using these or I will suggest removing this section from the manuscript.

Ans: Based on the reviewer's suggestion, this section was removed from the manuscript.

7. The authors predicted the discontinuous B-cell epitopes from the Vaccine construct, but they have not discussed the significance of these findings.

Ans: As per your valuable comment the above item were provided accordingly in the manuscript (line #576-580).

8. The authors have performed the MDS for the TLR-4 and Vaccine construct separately (Figure 10), but it was important to perform the MDS for the TLR4-vaccine complex for the checking the stability of the complex. The authors could perform a 100ns MDS instead of the 40ns to see the proper stability of the vaccine, TLR4 or the TLR4-vaccine complex.

Ans: Thanks for the your valuable comments. We investigated the behavior of the vaccine and the receptor in the docked complex, but their graphs were drawn separately and in some studies, it was done in the same way (DOI: [10.3390/vaccines10122010](https://doi.org/10.3390/vaccines10122010), DOI: [10.1038/s41598-022-11851-z](https://doi.org/10.1038/s41598-022-11851-z), DOI: [10.1016/j.ijbiomac.2020.07.117](https://doi.org/10.1016/j.ijbiomac.2020.07.117),DOI: [10.3389/fimmu.2022.865180](https://doi.org/10.3389/fimmu.2022.865180), DOI: [10.1016/j.sjbs.2021.06.082](https://doi.org/10.1016/j.sjbs.2021.06.082)), , but your comment is very interesting and constructive and will definitely be considered in future studies.

After 33 ns, the vaccine reached an almost steady state and TLR4 also fluctuates very little during the simulation period. Due to the lack of facilities, this part of the work was done by a bioinformatics company, and due to financial limitations, we could only pay the company for 40 ns. I am sure you are aware of the bad financial situation of my country (Iran) and I hope you understand me. In many studies, such as the following studies, the simulation time is considered to be less than 40 ns (even 10 ns).

DOI: [10.1038/s41598-022-14427-z](https://doi.org/10.1038/s41598-022-14427-z)

DOI: [10.1021/acsomega.9b00944](https://doi.org/10.1021/acsomega.9b00944)

DOI: [10.1007/s12026-022-09346-0](https://doi.org/10.1007/s12026-022-09346-0)

DOI: [10.1016/j.micpath.2020.104236](https://doi.org/10.1016/j.micpath.2020.104236)

DOI: [10.1016/j.imu.2020.100478](https://doi.org/10.1016/j.imu.2020.100478)

DOI: [10.1371/journal.pone.0244176](https://doi.org/10.1371/journal.pone.0244176)

DOI: [10.3390/vaccines10091564](https://doi.org/10.3390/vaccines10091564)

9. I would say the discussion section needs more improvement. In last few months, there are several research articles have been published for the multi-epitope vaccine design using immunoinformatic approaches in different journals. Could you please explain why this study is unique in comparison with the published articles.

Ans: Thank you so much for your constructive comment. This part was added to the discussion section(line #505-522, line # 545-547, line # 560-562, line # 566-569).

10. Figure legends need to be more descriptive.

Ans: Based on your valuable recommendation, Figure legends were modified.
